# d-Allulose Ameliorates Fructose-Induced Skeletal Muscle Insulin Resistance via Regulation of Ectopic Lipid Accumulation Independent of Body Weight Changes

**DOI:** 10.3390/nu17122050

**Published:** 2025-06-19

**Authors:** Shahriar Kamal, Yang Gou, Takamasa Tsuzuki, Linlin Fu, Takako Yamada, Ryoichi Banno, Teruhiko Koike

**Affiliations:** 1 Department of Sports Medicine, Graduate School of Medicine, Nagoya University, Nagoya 464-8601, Aichi, Japan; kamal.md.shahriar.b8@s.mail.nagoya-u.ac.jp (S.K.); fu.linlin.s9@s.mail.nagoya-u.ac.jp (L.F.);; 2 University of Washington Medicine Diabetes Institute, Department of Medicine, University of Washington, Seattle, WA 98109, USA; ygou@uw.edu; 3 Faculty of Pharmacy, Meijo University, Nagoya 468-8503, Aichi, Japan; ttsuzuki@meijo-u.ac.jp; 4 Research and Development, Matsutani Chemical Industry Co., Ltd., Itami 664-8508, Hyogo, Japan; takako-yamada@matsutani.co.jp; 5 Research Center of Health, Physical Fitness and Sports, Nagoya University, Nagoya 464-8601, Aichi, Japan

**Keywords:** d-allulose, high-fructose diet, insulin resistance, triglyceride accumulation

## Abstract

**Background/Objectives:** The consumption of fructose-sweetened beverages, especially when combined with a high-fat (HF) diet, substantially contributes to obesity, diabetes, and metabolic dysfunction-associated steatotic liver disease. Ectopic fat accumulation in skeletal muscles is a critical factor in the development of insulin resistance, a key feature of these metabolic disorders. We aimed to investigate the effects of the rare sugar, d-allulose, on fructose-induced insulin resistance. **Methods:** Male Wistar rats were randomly assigned to fructose-free control diet (CD), HF/fructose-free diet (HF), or HF/fructose diet (HFF) groups. After 4 weeks, an intraperitoneal glucose tolerance test (IPGTT) was performed, followed by a two-step hyperinsulinemic–euglycemic clamp (HE-clamp) test at 5 weeks. Blood, skeletal muscle, and liver samples were collected after 6 weeks, and triglyceride (TG) levels were measured. Additionally, Western blot was performed on skeletal muscle samples. The same protocol was repeated for the HFF group supplemented with either 5% d-allulose or 5% cellulose. **Results:** Compared to the CD and HF groups, the HFF group exhibited increased blood glucose levels during the IPGTT and greater systemic and skeletal muscle insulin resistance in the HE-clamp. Furthermore, plasma, liver, and muscle TG levels were significantly elevated in the HFF group. However, d-allulose supplementation improved insulin resistance in the HFF group and reduced blood, liver, and muscle TG levels. Additionally, insulin-stimulated AKT phosphorylation and acetyl-CoA carboxylase phosphorylation were enhanced in the skeletal muscle following d-allulose administration. **Conclusions:** d-allulose may improve insulin resistance by reducing TG accumulation in the skeletal muscle, potentially independent of its anti-obesity properties.

## 1. Introduction

The consumption of fructose-sweetened beverages, especially when combined with a high-fat (HF) diet, significantly contributes to obesity, diabetes, and metabolic dysfunction-associated steatotic liver disease. Insulin resistance, a central mechanism in the development of these conditions, arises through both obesity-dependent and obesity-independent pathways. For instance, aerobic exercise can acutely improve insulin sensitivity without altering body weight, with these beneficial effects lasting up to 72 h [1]. Moreover, while both HF and high-sugar diets promote visceral fat accumulation and obesity, they might induce insulin resistance via distinct mechanisms that extend beyond the effects of obesity alone [2].

The interplay between lipids and carbohydrates is critical in the development of insulin resistance. Many diet-induced obesity studies have employed high-sucrose or HF diets to model type 2 diabetes. However, HF diets often contain substantial amounts of sugar, making it difficult to isolate the effects attributable solely to fat. Furthermore, HF diets might only induce insulin resistance when consumed in excessive quantities [3,4]. Similarly, studies investigating high-sucrose and high-fructose diets have often used conditions where these carbohydrates constitute over 50% of total energy intake [5,6,7]. Given that typical human fructose consumption does not exceed 15% of total energy intake [8], the interpretation of these studies warrants caution. To address these limitations, our study utilized a diet specifically designed to investigate the effects of fructose on insulin resistance.

d-allulose, a rare sugar and a C-3 epimer of fructose, has been recognized as safe by the United States Food and Drug Administration and is used as a sweetener and dietary supplement [9]. Human studies have indicated its potential in lowering blood glucose levels and reducing obesity [10,11,12,13]. d-allulose appears to improve glucose control by enhancing pancreatic beta cell function, increasing glucokinase activity in the liver, and reducing fat accumulation and inflammatory responses [14]. Our previous research demonstrated that d-allulose improves insulin resistance in HF [15] and high-sucrose obesity rat models [16] using the insulin clamp method. Additionally, we found that d-allulose increased insulin sensitivity and exercise capacity in normal mice, mirroring the effects of exercise [17,18].

In our prior studies using HF and high-sucrose obesity models, it remained unclear whether the beneficial effects of d-allulose on insulin resistance were due to its anti-obesity or direct metabolic actions. Notably, d-allulose seemed to elicit greater improvements in insulin sensitivity in the high-sucrose obesity model compared to the HF model, suggesting a potential interaction with sugar metabolism.

Therefore, the present study aimed to specifically investigate the role of dietary fructose in inducing insulin resistance in skeletal muscle. Furthermore, we sought to elucidate the mechanisms by which d-allulose mitigates insulin resistance, focusing on effects independent of body weight changes. Considering that d-allulose is a C-3 epimer of fructose, we hypothesized that d-allulose selectively counteracts fructose-induced metabolic disturbances.

## 2. Materials and Methods

### 2.1. Rats and Diets

A total of 130 six-week-old male Wistar rats (mean ± SD body weight: 161.8 ± 7.1 g) were purchased from Japan SLC, Inc. (Shizuoka, Japan) and individually housed in plastic cages (38.4 × 33.5 × 17.3 cm; L × W × H) with paper bedding (Palmas μ; Material Research Center, Kanagawa, Japan). Rats had ad libitum access to food and water. The housing environment was maintained at a constant temperature of 22 ± 2 °C under a 12:12 h light–dark cycle (8 a.m. to 8 p.m.). Following a 1-week acclimatization period, the rats were randomly assigned to three groups receiving distinct diets: a fructose-free control diet (CD), a fructose-free HF diet (HF), and an HF/fructose diet (HFF). The Match Purina 5053 fructose-free control (D17011901), HF (D19090601), and HFF (D22092104) diets were sourced from Research Diets Inc. (New Brunswick, NJ, USA). The detailed dietary profiles are presented in Table S1. To investigate the effects of d-allulose (Matsutani Chemical Industry Co., Ltd., Hyogo, Japan), the HFF rats were further randomized into two subgroups receiving either 5% (*w*/*w*) d-allulose or cellulose (Oriental Yeast Co., Ltd., Tokyo, Japan). All experimental procedures adhered to the Guidelines for the Care and Use of Laboratory Animals of Nagoya University, and ethical approval was granted by the Animal Experiment Committee of Nagoya University (H250003-002). Rats were euthanized by an overdose of pentobarbital sodium (200 mg/kg body weight), administered intravenously. If intravenous access was not feasible, the drug was administered intraperitoneally. Rats were euthanized following the completion of the hyperinsulinemic–euglycemic (HE) clamp test.

The sample size was determined based on our previous studies using similar rat models [15,16], which indicated that a group size of approximately 5 to 10 rats consistently yielded statistically significant differences in HE-clamp and intraperitoneal glucose tolerance tests (IPGTT). In total, 130 rats were used in this study: 30 rats were assigned to each of the CD, HF, and HFF groups, while 20 rats were assigned to each of the HFF supplemented with cellulose (HFF-C) and HFF supplemented with d-allulose (HFF-A) groups. In the CD, HF, and HFF groups, 15 rats underwent the HE-clamp and IPGTT, and the other 15 rats were used for tissue sampling. In the HFF-C and HFF-A groups, 10 rats underwent the HE-clamp and IPGTT, and the other 10 rats were used for tissue sampling. The HE-clamp and IPGTT experiments were independently repeated at least twice, and the representative results are presented. The number of rats included in statistical analyses for each experiment is specified in the corresponding figure legends.

### 2.2. Experimental Protocols

#### 2.2.1. Hyperinsulinemic Euglycemic (HE)-Clamp Test

Following 4 weeks of dietary intervention, the rats underwent cannulation, with the HE-clamp test performed 5 days post-surgery. The cannulation procedure and HE-clamp test were conducted as previously detailed [19,20]. After the 4-week feeding period, the rats were anesthetized via intraperitoneal injection of a combination of three anesthetics: medetomidine (0.15 mg/kg), midazolam (2 mg/kg), and butorphanol (2.5 mg/kg), prior to the surgical procedure. Catheters were inserted into the right jugular vein for the infusion of a 20% glucose solution and insulin, and into the left carotid artery for blood sampling. The HE-clamp test was performed 5 days after surgery, ensuring the rats had recovered and their body weight reduction was less than 10% of their pre-operative weight. Prior to the HE-clamp test, the rats were fasted for 16 h. During the test, the rats were housed in comfortable cages with unrestricted movement. A continuous two-step clamp protocol was employed. In the first step, a low-dose insulin infusion (3 mU/kg BW/min) was administered for 120 min. Subsequently, a high-dose insulin infusion (30 mU/kg BW/min) was administered for another 120 min. Blood glucose levels were measured at 10 min intervals. The glucose infusion rate was adjusted throughout the clamp to maintain euglycemia at 90 mg/dL. Steady-state was defined when the range of last three consecutive blood glucose measurements was less than or equal to 18 mg/dL. The glucose infusion rate (GIR) achieved at steady state was used as an indicator of insulin resistance.

#### 2.2.2. Intraperitoneal Glucose Tolerance Test (IPGTT) and Blood, Liver, and Muscle Sampling

Following 4 weeks of dietary intervention, an IPGTT was performed. The rats were fasted for 6 h prior to the test. A bolus of 20% glucose solution (2 g/kg BW) was administered via intraperitoneal injection. Blood samples for glucose level assessment were collected from the tail vein at the following time points: T = 0 (before glucose injection) and 30, 60, 90, and 120 min post-injection. The blood samples collected at 0 and 30 min were used for insulin measurements. After 6 weeks of dietary intervention, blood, skeletal muscle, and liver samples were collected as detailed below.

#### 2.2.3. Blood Glucose and Plasma Insulin

Blood samples were collected from the tail tip incision. Blood glucose levels were measured using a NIPRO Stat Strip XP3 (NIPRO Co., Ltd., Osaka, Japan). Plasma insulin levels were quantified using an ELISA kit (FUJIFILM Wako Sibayagi Corporation, Gunma, Japan) and a Multiskan FC microplate reader (Thermo Fisher Scientific Inc., Waltham, MA, USA). Insulin resistance was estimated using the Homeostatic Model Assessment for Insulin Resistance (HOMA-IR), calculated with the following equation:

HOMA-IR = Fasting insulin (pg/mL)/38.5 × fasting glucose (mg/dL)/405(1)

#### 2.2.4. Intracellular Signaling Analysis in Skeletal Muscle Using Western Blotting

After 6 weeks of their respective dietary interventions, the rats were anesthetized via the intraperitoneal injection of medetomidine (0.15 mg/kg), midazolam (2 mg/kg), and butorphanol (2.5 mg/kg). Subsequently, insulin (0.5 IU/kg) was administered through the inferior vena cava, and the gastrocnemius muscle was rapidly excised after 2 min, followed by the excision of the liver, which resulted in death by exsanguination. Because the rats were maintained under adequate anesthesia during the procedure, they did not regain consciousness thereafter. Fat tissues were isolated postmortem. The harvested muscle samples were immediately snap-frozen in liquid nitrogen and stored at −80 °C for further analysis.

Western blotting was performed as previously described [21]. Briefly, 30 µg protein extracts from the muscle tissue were separated by SDS-PAGE and then transferred onto polyvinylidene difluoride membranes (Merck Millipore, Darmstadt, Germany). The membranes were blocked for 1 h at room temperature (20–25 °C) using 5% nonfat milk in TBS-T buffer (20 mM Tris, pH 7.6; 0.8% NaCl; and 0.1% Tween 20). Following the blocking step, the membranes were incubated for 1 h at room temperature with the following primary antibodies: Phospho-Akt (Ser473) #9271, AMP-activated protein kinase (AMPK)α #2532, Phospho-AMPKα (Thr172) #2531, acetyl-CoA carboxylase (ACC; C83B10) #3676, Phospho-ACC (Ser79) #3661 (all from Cell Signaling Technology, Inc., Danvers, MA, USA); and Akt1/2/3 (5C10); sc-81434 (Santa Cruz Biotechnology, Inc., Santa Cruz, CA, USA). After incubation with the primary antibodies, the membranes were washed five times for 5 min each with TBS-T and then incubated for 1 h at room temperature with a 1:1000 dilution of either horseradish peroxidase-conjugated goat anti-rabbit (Bio-Rad, Laboratories Inc., Hercules, CA, USA) or anti-mouse IgG antibody (KPL, Gaithersburg, MD, USA). The membranes were again washed five times with TBS-T. Protein bands were visualized using ECL reagent (GE Healthcare UK Ltd., Buckinghamshire, UK). Images of each membrane were captured using a Luminograph I imaging system (ATTO Co., Ltd., Tokyo, Japan) and quantified using ImageJ software 1.47v (National Institutes of Health, Bethesda, MD, USA).

Individual data points from the control group (cellulose-fed HFF group) were divided by the group mean. Consequently, the mean of the normalized control group was 1 with associated variability. The density of the protein band for the d-allulose-fed HFF group was expressed as the fold change in density relative to the cellulose-fed HFF group values.

#### 2.2.5. Triglyceride (TG) Measurement

Following 6 weeks of the respective dietary interventions, plasma, muscle, and liver TG levels were measured as previously described [22]. For plasma TG measurements, blood samples were collected from the tail vein after a 3 h fasting period at the end of the dark cycle (8 am). Plasma was separated and stored in aliquots at −80 °C. To determine liver and muscle TG levels, tissue samples were crushed and homogenized in a buffer containing 5% (*v*/*v*) NP-40 substitute (Fujifilm Wako, Osaka, Japan). The resulting homogenate underwent two heating cycles (5 min at 100 °C) with intermittent cooling to room temperature to facilitate lipid solubilization. After centrifugation at 10,000× *g* for 2 min, the TG content of the supernatant was quantified using the LabAssay Triglyceride Kit (Fujifilm Wako, Osaka, Japan).

### 2.3. Statistical Analysis

Data are presented as mean ± SD. Statistical significance was defined as *p* < 0.05. Data from the longitudinal studies were analyzed using two-way ANOVA with repeated measures, followed by Tukey’s multiple comparison test to identify specific differences. When a significant group–time interaction was observed, between-group comparisons were conducted at each time point. Comparisons involving two groups were performed using Student’s *t*-test, while comparisons among three groups were conducted using one-way ANOVA followed by Tukey’s multiple comparison test. Pearson’s correlation coefficient was used to determine the association between two groups. All statistical analyses were performed using GraphPad Prism version 6.07 (GraphPad Software Inc., San Diego, CA, USA).

## 3. Results

### 3.1. Increased Visceral Fat but Not Body Weights in the HFF Group

**Figure 1:**
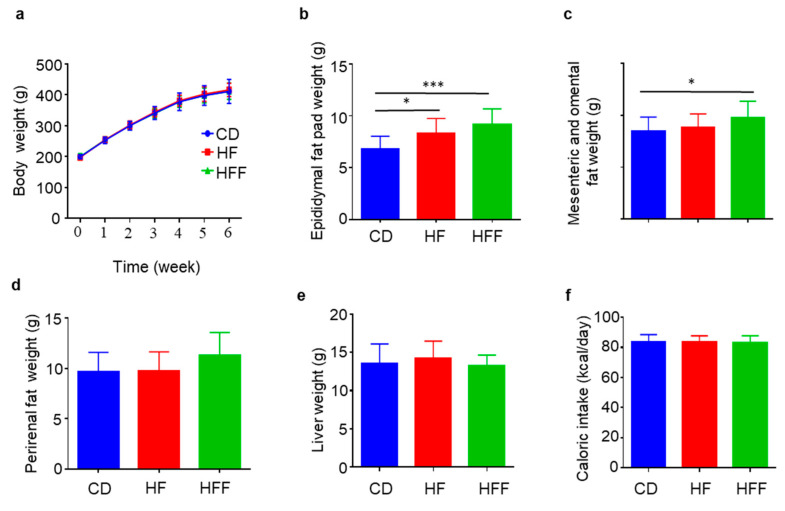
Body and fat weights after 6 weeks of dietary intervention. (**a**) Body weight, (**b**) epididymal fat pad weight, (**c**) mesenteric and omental fat weight, (**d**) perirenal fat weight, (**e**) liver weight, and (**f**) caloric intake. Results are expressed as mean ± SD; * *p* < 0.05, *** *p* < 0.005, (**a**–**d**,**f**) *n* = 15, (**e**) *n* = 9. Statistical differences were determined using two-way ANOVA for repeated measures (**a**) and one-way ANOVA (**b**–**f**). CD: control diet, HF: high-fat diet, and HFF: high-fat/fructose diet.

After 6 weeks of dietary intervention, there was no significant difference in body weight among the CD, HF, and HFF groups (Figure 1a). However, the epididymal fat pad weight was significantly greater in both the HF and HFF groups compared to the CD group (Figure 1b). Mesenteric and omental fat weights were significantly increased in the HFF group compared to the CD group, but not in the HF group (Figure 1c). In contrast, the perirenal fat weight, a type of fat distinct from visceral fat, remained comparable across all three groups (Figure 1d). Similarly, liver weight (Figure 1e) and average daily caloric intake (kcal/day) (Figure 1f) did not differ significantly among the groups. Food consumption (g/day) was higher in the CD group compared to the HF and HFF groups after the initial 2 days of feeding each respective diet (Figure S1a). Conversely, a significant difference in caloric intake per day was observed among the groups during the first 2 days, after which caloric intake became similar (Figure S1b). The feed efficiency ratio was also comparable between the groups (Figure S1c).

### 3.2. Elevated Blood Glucose Levels During IPGTT in the HFF Group

**Figure 2:**
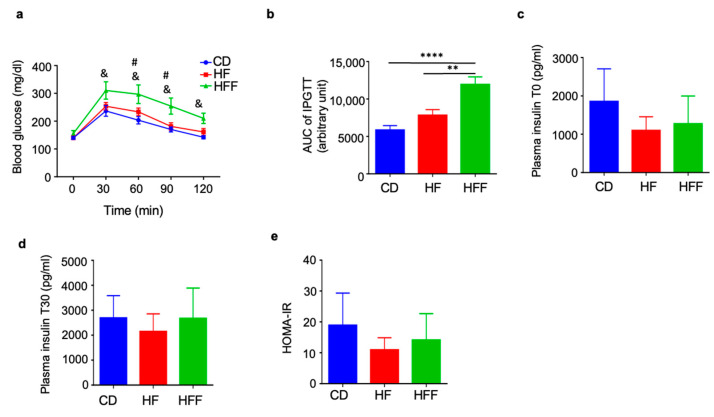
Elevated blood glucose in IPGTT after 4 weeks of fructose administration. (**a**) Blood glucose, (**b**) area under the curve, (**c**) basal insulin levels, (**d**) insulin levels at 30 min post-glucose injection, and (**e**) HOMA-IR. Results are expressed as mean ± SD; #: HF vs. HFF, &: CD vs. HFF. ^#,&^ *p* < 0.05; ** *p* < 0.01, **** *p* < 0.01, (**a**–**e**) *n* = 8 per group. Statistical differences were determined using two-way ANOVA for repeated measures (**a**) and one-way ANOVA (**b**–**e**). CD: control diet, HF: high-fat diet, HFF: high-fat/fructose diet.

The IPGTT demonstrated a significant increase in blood glucose levels in the HFF group compared to both the CD and HF groups (Figure 2a,b). In contrast, basal insulin levels, insulin levels at 30 min post-glucose injection, and the HOMA-IR index did not show significant differences among the three groups (Figure 2c–e).

### 3.3. Muscle and Systemic Insulin Resistance in HE-Clamp Test

**Figure 3:**
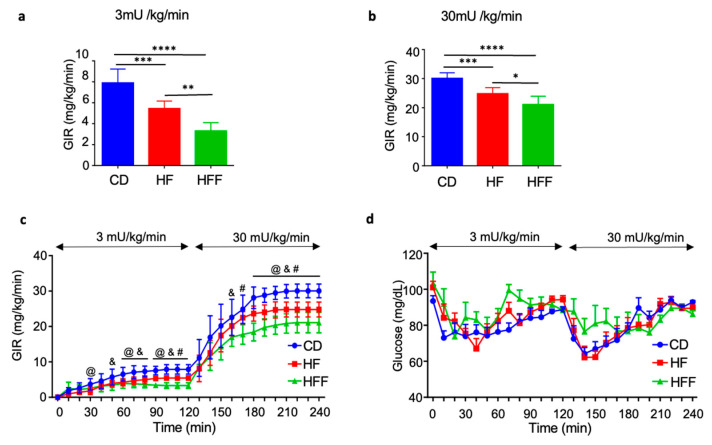
Changes in glucose infusion rate (GIR) after 5 weeks of dietary intervention during the hyperinsulinemic euglycemic clamp test: (**a**) GIR at low-dose insulin infusion (3 mU/kg/min), (**b**) GIR at high-dose insulin infusion (30 mU/kg/min), (**c**) the time course of GIR throughout the clamp procedure, and (**d**) the corresponding time course of blood glucose levels. Low- and high-dose insulin were continuously infused during the first (0–120 min) and second (120–240 min) steps, respectively. Results are expressed as mean ± SD; @: CD vs. HF, #: HF vs. HFF, &: CD vs. HFF. ^@,#,&^ *p* < 0.05; * *p* < 0.05, ** *p* < 0.01, *** *p* < 0.005, **** *p* < 0.001, (**a**–**d**) *n* = 8 (CD), 7 (HF), 8 (HFF). Statistical differences were determined using one-way ANOVA (**a**,**b**) and one-way ANOVA at each time point (**c**). CD: control diet, HF: high-fat diet, HFF: high-fat/fructose diet.

To assess insulin resistance, we performed a two-step HE-clamp test. During both the low-dose insulin infusion (3 mU/kg/min; Figure 3a) and the high-dose insulin infusion (30 mU/kg/min; Figure 3b), the GIR was significantly lower in the HF group than in the control group. Furthermore, the GIR was even lower in the HFF group than in the HF group. The reduced GIR observed during the low-dose clamp procedure indicates systemic insulin resistance, encompassing both liver and skeletal muscle insulin resistance. Conversely, the decreased GIR during the high-dose clamp primarily reflects skeletal muscle insulin resistance. The specific GIR values and corresponding glucose levels achieved under both low- and high-dose insulin fusion conditions are presented in Figure 3c and Figure 3d, respectively.

### 3.4. Changes in Blood, Muscle, and Liver TG

**Figure 4:**
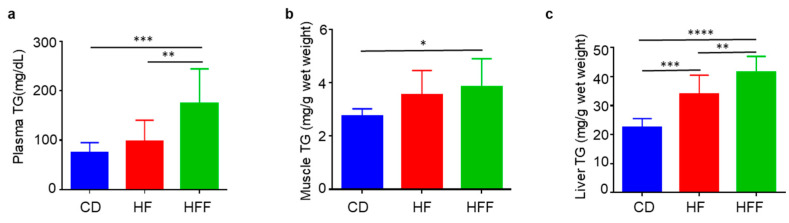
Changes in blood, muscle, and liver triglyceride (TG) levels after 6 weeks of dietary intervention. (**a**) Plasma TG, (**b**) muscle TG, and (**c**) liver TG. Results are expressed as mean ± SD; * *p* < 0.05, ** *p* < 0.01, *** *p* < 0.005, **** *p* < 0.001, (**a**–**c**) *n* = 10 per group. Statistical differences were determined using a one-way ANOVA. CD: control diet, HF: high-fat diet, HFF: high-fat/fructose diet.

Plasma, muscle, and liver TG levels were significantly elevated in the HFF diet group than in the CD diet group (Figure 4a–c). Furthermore, plasma and liver TG levels were significantly higher in the HFF diet group than in the HF diet group (Figure 4a,c). Notably, the liver TG levels were also higher in the HF group than in the CD group (Figure 4c).

### 3.5. Effect of d-Allulose on Body Weights and Fat Weights in the HFF Group

**Figure 5:**
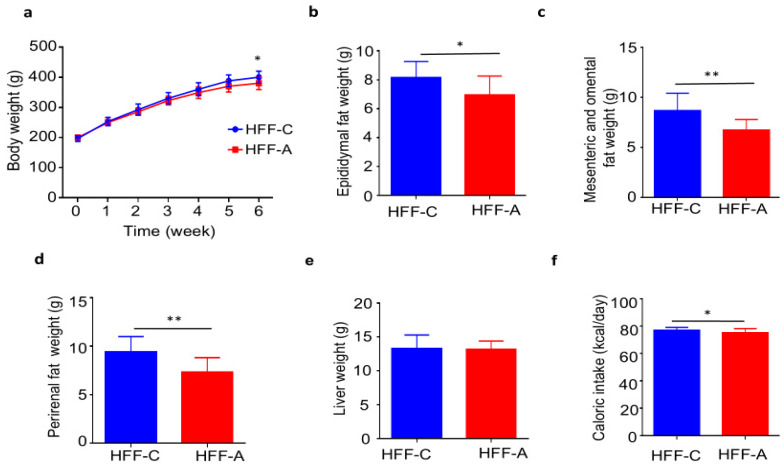
Effect of d-allulose administration on body weight and fat mass. (**a**) Body weight, (**b**) epididymal fat pad weight, (**c**) mesenteric and omental fat weight, (**d**) perirenal fat weight, (**e**) liver weight, and (**f**) caloric intake. Results are expressed as mean ± SD; * *p* < 0.05, ** *p* < 0.01, (**a**–**f**) *n* = 10 per group. Statistical differences were determined using two-way ANOVA for repeated measures (**a**) and unpaired *t*-tests (**b**–**f**). HFF-C: high-fat/fructose-cellulose, HFF-A: high-fat/fructose-d-allulose.

Body weights remained similar between the d-allulose-fed and cellulose-fed groups for the initial 5 weeks. However, a significant difference in body weight emerged at week 6 following the administration of d-allulose (Figure 5a). The weights of visceral fat depots, including the epididymal fat pad and mesenteric/omental fat (Figure 5b,c), as well as perirenal fat (Figure 5d), were significantly lower in the d-allulose-fed group than in the cellulose-fed group. Liver weights were comparable between the two groups (Figure 5e). Notably, the average calorie intake was significantly reduced in the d-allulose-fed group than in the cellulose-fed group. Food consumption (g/d) is illustrated in Figure S1d and the feed efficiency ratio (g/kcal) was lower in the d-allulose-fed group (Figure S1e).

### 3.6. Effect of d-Allulose on Blood Glucose in IPGTT in the HFF Group

**Figure 6:**
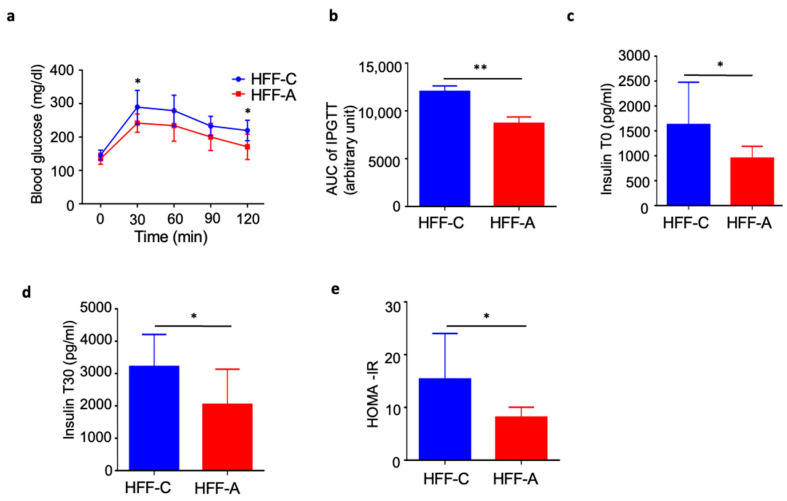
Effect of d-allulose administration on glucose changes in IPGTT. (**a**) Blood glucose levels during IPGTT, (**b**) area under the curve during IPGTT, (**c**) basal insulin levels, (**d**) insulin levels at 30 min post-glucose injection, and (**e**) HOMA-IR. Results are expressed as mean ± SD; * *p* < 0.05, ** *p* < 0.01, (**a**–**e**) *n* = 8, per group. Statistical differences were determined using two-way ANOVA for repeated measures (**a**) and unpaired *t*-test (**b**–**e**). HFF-C: high-fat/fructose-cellulose, HFF-A: high-fat/fructose-d-allulose.

The IPGTT demonstrated a significant reduction in blood glucose levels in the d-allulose-fed HFF group than in the cellulose-fed HFF group (Figure 6a,b). Furthermore, basal insulin levels, insulin levels at 30 min post-glucose injection, and the HOMA-IR index were significantly lower in the d-allulose-fed HFF group than in the cellulose-fed HFF group (Figure 6c–e).

### 3.7. Effect of d-Allulose on Insulin Resistance in the HFF Group

**Figure 7:**
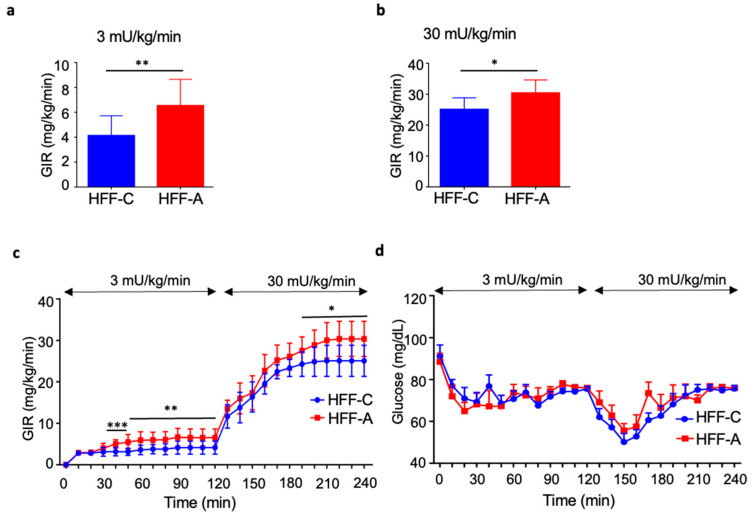
Effect of d-allulose administration on glucose infusion rate (GIR) in the hyperinsulinemic–euglycemic clamp test. (**a**) GIR at low-dose insulin (3 mU/kg/min), (**b**) GIR at high-dose insulin (30 mU/kg/min), (**c**) the time course of GIR, and (**d**) the time course of blood glucose. Low- and high-dose insulin were continuously infused during the first (0–120 min) and the second (120–240 min) steps, respectively. Results are expressed as mean ± SD; * *p* < 0.05, ** *p* < 0.01, *** *p* < 0.005, (**a**) *n* = 10 per group, (**b**) *n* = 8 per group, (**c**,**d**) 3 mU/kg/min: *n* = 10 per group, 30 mU/kg/min: *n* = 8 per group. Statistical differences were determined using an unpaired *t*-test (**a**,**b**) and an unpaired *t*-test at each time point (**c**). HFF-C: high-fat/fructose-cellulose, HFF-A: high-fat/fructose-d-allulose.

A two-step HE-clamp test demonstrated that the GIR was significantly higher in the d-allulose-fed HFF group than in the cellulose-fed HFF group under both low-dose (3 mU/kg/min; Figure 7a) and high-dose (30 mU/kg/min; Figure 7b) insulin infusion conditions. Notably, our findings indicate that d-allulose administration improved insulin resistance, specifically in the skeletal muscles. The detailed GIR values and corresponding glucose levels during the low- and high-dose clamp procedures are presented in Figure 7c and Figure 7d, respectively.

### 3.8. Effect of d-Allulose on Blood, Muscle, and Liver TG in the HFF Group

**Figure 8:**
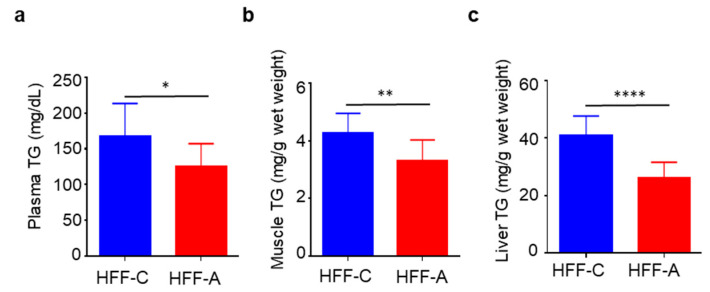
Effect of d-allulose administration on blood, muscle, and liver triglyceride (TG) after 6 weeks of dietary intervention. (**a**) Plasma, (**b**) muscle, and (**c**) liver TG. Results are expressed as mean ± SD; * *p* < 0.05, ** *p* < 0.01, **** *p* < 0.001, (**a**–**c**) *n* = 10 per group. Statistical differences were determined using an unpaired *t*-test. HFF-C: high-fat/fructose-cellulose, HFF-A: high-fat/fructose-d-allulose.

Plasma, muscle, and liver TG levels were significantly lower in the d-allulose-fed HFF group compared to the cellulose-fed HFF group (Figure 8a–c). The correlation between body weight and TG levels is shown in Figure S3, indicating that body weight was unlikely to have influenced these results.

### 3.9. Effect of d-Allulose on Signaling Molecules in Skeletal Muscle

**Figure 9:**
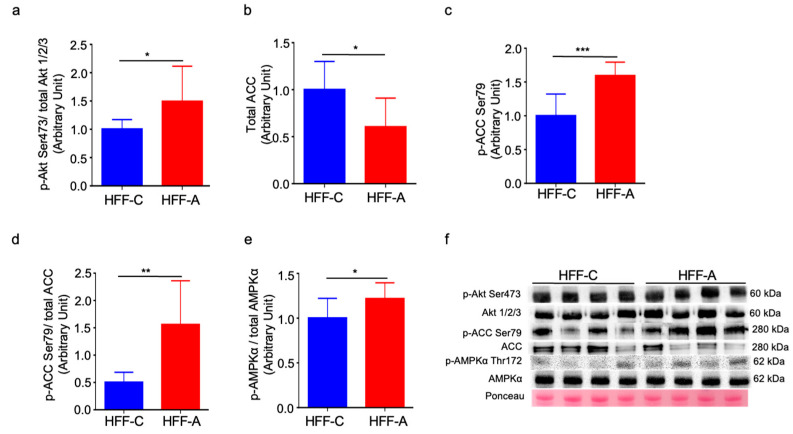
Effect of d-allulose administration on expression and phosphorylation of AKT, ACC, and AMPK in gastrocnemius muscle. (**a**) Phosphorylation of AKT at Ser473, (**b**) expression and (**c**,**d**) phosphorylation of ACC at Ser79, (**e**) phosphorylation of AMPKα at Thr172, and (**f**) representative Western blot images. Results are expressed as mean ± SD; * *p* < 0.05, ** *p* < 0.01, *** *p* < 0.005, (**a**–**e**) *n* = 8 per group. Statistical differences were determined using an unpaired *t*-test. HFF-C: high-fat/fructose-cellulose, HFF-A: high-fat/fructose-d-allulose.

Insulin-stimulated phosphorylation of Akt at Ser473 was significantly elevated in the d-allulose-fed HFF group compared to the cellulose-fed HFF group (Figure 9a), indicating an improvement in skeletal muscle insulin sensitivity. The expression of ACC was significantly lower while its phosphorylation at Ser79 was significantly higher in the d-allulose fed HFF group than in the cellulose-fed HFF group (Figure 9b–d). Furthermore, the phosphorylation of AMPKα at Thr172 was significantly higher in the d-allulose-fed HFF group than in the cellulose-fed HFF group (Figure 9e). Representative Western blot images are shown in Figure 9f, with their corresponding uncropped images available in Figure S2.

## 4. Discussion

Using the two-step HE clamp method, we observed that d-allulose administration reversed fructose-induced skeletal muscle insulin resistance. This improvement appears to be mediated, at least in part, by a reduction in ectopic fat deposition within the skeletal muscle and occurs independently of its anti-obesity effects. Although our previous work, which used high-dose insulin infusion (supraphysiological insulin levels) in HE clamp experiments, suggested a skeletal muscle-specific increase in insulin resistance, this study, for the first time, indicates the potential mechanism behind muscle-specific insulin resistance—ectopic fat deposition. As an epimer of fructose, d-allulose may counteract fructose-induced insulin resistance through distinct mechanisms. To delineate the specific roles of fat and fructose in the development of insulin resistance, we compared the metabolic consequences of an HF diet and an HFF diet, using a fructose-free low-fat diet as a control. The subsequent analysis of the HFF-fed rats treated with d-allulose revealed that it almost completely normalized fat mass, glucose metabolism, insulin sensitivity, and triglyceride levels to those observed in the CD group, indicating a strong modulatory effect on sugar metabolism.

d-allulose has demonstrated an antihyperglycemic effect in humans [10]. As a rare, zero-calorie sugar, it has been investigated as a functional sweetener with potential anti-obesity properties. Its metabolic benefits have been attributed to mechanisms such as the suppression of proinflammatory cytokines, reduced intestinal absorption of glucose and fructose, and improved insulin resistance [14]. These effects can arise from both the direct action of d-allulose and its secondary impact on body weight reduction. However, in contrast to our previous studies where weight loss significantly contributed to improvements in insulin resistance [15,16], the beneficial effects of d-allulose observed in this study were largely independent of its anti-obesity action.

Previous research has indicated that d-allulose can decrease appetite and body weight [23,24,25,26], leading to reduced food consumption and body weight loss as early as the first week of administration. Conversely, in the present study, no significant differences in body weight between the d-allulose and control groups were observed until week 6. Notably, body weights remained similar among the HFF, HF, and CD groups throughout most of the study, suggesting that differences in body weight were not a primary factor in fructose-induced insulin resistance in our model. Furthermore, while significant weight loss was observed in the HFF group in the sixth week of d-allulose administration, a significant improvement in IPGTT was evident as early as week 4. These findings strongly suggest that the beneficial effects of d-allulose on insulin resistance occur independently of changes in body weight.

We also investigated the specific role of fructose within the context of HF feeding. Although reducing saturated fatty acid intake is commonly recommended, its role in the development of type 2 diabetes remains a subject of debate. A review of prospective human and clinical trials found no clear evidence that moderate amounts of dietary saturated fatty acids increase the risk of insulin resistance or type 2 diabetes [3]. Indeed, HF diets alone are not consistently associated with increased insulin resistance or cardiovascular disease. Even in low-carbohydrate diets with a high fat-to-energy ratio (70%), insulin resistance levels remained stable, regardless of whether the fat source was saturated fatty acids or eicosapentaenoic acid [4]. In our study, the HF diet contained 40% fat but was not low-carbohydrate, utilizing starch as the primary carbohydrate and excluding sucrose and fructose. The IPGTT results showed that glucose levels remained comparable between the CD and HF groups, but were significantly elevated in the HFF group. Similarly, the HE-clamp results indicated a significant increase in insulin resistance in the HFF group, whereas the HF group exhibited only a modest increase, possibly attributable to liver TG accumulation. Notably, muscle TG levels remained similar between the HF and CD groups, which may explain the lack of change in glucose tolerance between these groups in the IPGTT.

Fructose, a prevalent dietary sugar, is strongly implicated in adverse metabolic outcomes. Several mechanisms may contribute to these effects. First, Taylor et al. demonstrated that dietary fructose enhances intestinal cell survival and increases intestinal villus length, potentially leading to greater nutrient absorption and adiposity [27]. Second, fructose metabolism is closely linked to liver steatosis, and inhibiting fructose metabolism has been shown to alter metabolic outcomes [28]. While TG accumulation is a recognized contributing factor, it does not fully account for fructose-induced insulin resistance. Softic et al. demonstrated that although both fructose and glucose contribute to liver lipid accumulation, only fructose leads to obesity, glucose tolerance, and hepatomegaly [29]. Finally, the co-ingestion of fructose and TGs can induce postprandial lipidemia by impairing TG clearance [30]. The present study demonstrated that targeting fructose metabolism is a key mechanism by which d-allulose ameliorates diet-related metabolic disturbances, including those induced by high-fat and high-sucrose intake.

We propose two potential mechanisms by which d-allulose administration reduces muscle TG levels. First, as an epimer of fructose, d-allulose may competitively inhibit fructose absorption in the intestine, thereby reducing TG accumulation [31]. A reduction in intestinal TG absorption by d-allulose has also been proposed [32]. Second, d-allulose, being calorie-free, may enhance fatty acid oxidation. Previous studies have reported increased fatty acid oxidation following d-allulose administration [33,34,35], possibly through increased ACC phosphorylation, which promotes fatty acid oxidation in muscles [36]. AMPK, a sensor of cellular energy status, induces ACC phosphorylation upon activation. d-allulose, with its potential to modulate glycolysis and enhance fatty acid oxidation, has been proposed as a caloric restriction mimetic [37,38].

Oxidative stress, endoplasmic reticulum stress, and chronic inflammation have been implicated in skeletal muscle insulin resistance and may interact through complex intracellular signaling pathways [39]. In addition, lipid intermediates such as diacylglycerols or ceramides may play critical roles in the development of insulin resistance [39,40,41]. Accordingly, further studies are necessary to elucidate the detailed mechanisms; however, our findings suggest that d-allulose ameliorates insulin resistance by reducing muscle fat accumulation, independent of its effects on weight loss.

The limitations of this study include the exclusive use of male Wistar rats and the absence of analyses across different skeletal muscle types. These constraints underscore the need for further mechanistic studies to elucidate the precise pathways through which d-allulose improves skeletal muscle insulin resistance. Although the impact of sex differences on metabolism is important, the key mechanisms by which d-allulose acts in our study—the inhibition of fructose absorption, reduction in ectopic lipid accumulation, and activation of AMPK signaling—are not expected to differ by sex. Furthermore, while the dosages administered in animal studies are relatively high compared to typical human consumption, beneficial effects have already been demonstrated at comparatively low doses in humans, including males and females. Concomitant factors such as dietary composition, timing of intake, and physical activity can also influence the effect of d-allulose. Considering the widespread consumption of fructose-rich sweetened beverages, clinical studies are warranted to determine whether d-allulose can effectively counteract fructose-induced insulin resistance and ectopic lipid accumulation in humans.

## 5. Conclusions

**Figure 10:**
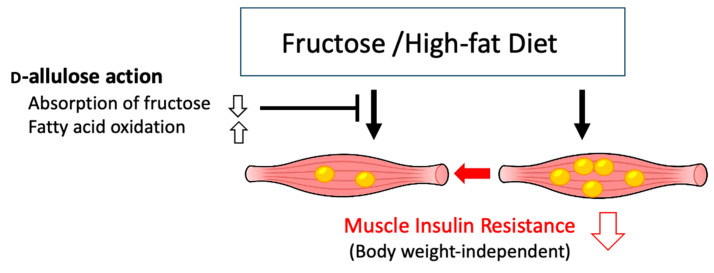
Proposed action of d-allulose on skeletal muscle insulin resistance.

This study provides new insights into the metabolic effects of fructose and suggests that d-allulose is a promising agent for improving insulin sensitivity in skeletal muscles without necessitating weight loss. To the best of our knowledge, this is the first study to demonstrate that d-allulose specifically counteracts fructose-induced skeletal muscle insulin resistance by reducing ectopic lipid accumulation, independent of its anti-obesity properties (Figure 10).

## Data Availability

The original contributions presented in the study are included in the article/Supplementary Materials, further inquiries can be directed to the corresponding author.

## Supplementary Materials

The following supporting information is available for download at https://www.mdpi.com/article/10.3390/nu17122050/s1, including Table S1: Customized diet composition; Figure S1: Food consumption and feed efficiency ratios; Figure S2: Uncropped images of Western blot; Figure S3: Relationship between body weight and plasma, muscle, or liver TG.

## Abbreviations

The following abbreviations are used in this manuscript:

CD	Control diet
GIR	Glucose infusion rate
HF	High-fat
HFF	High-fat/fructose
HFF-A	High-fat/fructose-d-allulose
HFF-C	High-fat/fructose-cellulose
IPGTT	Intraperitoneal glucose tolerance test
TG	Triglyceride
