# Supplementary material for: d-Allulose Ameliorates Fructose-Induced Skeletal Muscle Insulin Resistance via Regulation of Ectopic Lipid Accumulation Independent of Body Weight Changes
Source: Nutrients. 2025 Jun 19;17(12):2050. doi: 10.3390/nu17122050 (PMC12196061; doi:10.3390/nu17122050)
Supplement: Supplementary file 1 [file nutrients-17-02050-s001.zip › nutrients-3660362-supplementary.pdf]

Supplementary Materials

Table S1: Custom diet compositions of control fructose-free, high-fat fructose-free, and high-fat/fructose diets.

Product	D17011901 (Matcha Purnia 5053)		D19090601		D22092104	
	Control Diet (CD)		High-fat Diet (HF)		High-fat /fructose Diet (HFF)	
	gm%	Kcal%	gm%	Kcal%	gm%	Kcal%
Protein	23.4	25.0	28.0	25.0	28.0	25.0
Carbohydrate	59.9	62.0	36.0	30.0	36.0	30.0
Fat	5.4	13.0	22.4	45.0	22.4	45.0
Total		100		100		100
kcal/gm	3.76		4.51		4.51	
**Ingredient**	**gm**	**kcal**	**gm**	**kcal**	**gm**	**kcal**
Casein	250	1000	250	1000	250	1000
L-Cystine	3.8	15.2	3.8	15.2	3.8	15.2
Corn Starch	381	1524	57	228	0	0
Maltodextrin 10	110	440	110	440	0	0
Sucrose	0	0	0	0	0	0
Fructose	0	0	0	0	291	1164
Dextrose	124	496	124	496	0	0
Cellulose, BW200	75	0	75	0	75	0
Inulin	25	37.5	25	37.5	25	37.5
Soyabean Oil	59	531	59	531	59	531
Lard	0	0	144	1296	144	1296
Mineral Mix S10026	10	0	10	0	10	0
Dicalcium Phosphate	13	0	13	0	13	0
Calcium Carbonate	5.5	0	5.5	0	5.5	0
Potassium Citrate, 1 H2O	16.5	0	16.5	0	16.5	0
Vitamin Mix V10001	10	40	10	40	10	40
Choline Bitartrate	2	0	2	0	2	0
Yellow Dye # 5, FD&C	0.025	0	0.05	0	0	0
Red Dye #40. FD&C	0	0	0	0	0	0
Blue Dye #1, FD&C	0.025	0	0	0	0.05	0
**Total**	**1084.85**	**4084**	**904.85**	**4084**	**904.85**	**4084**

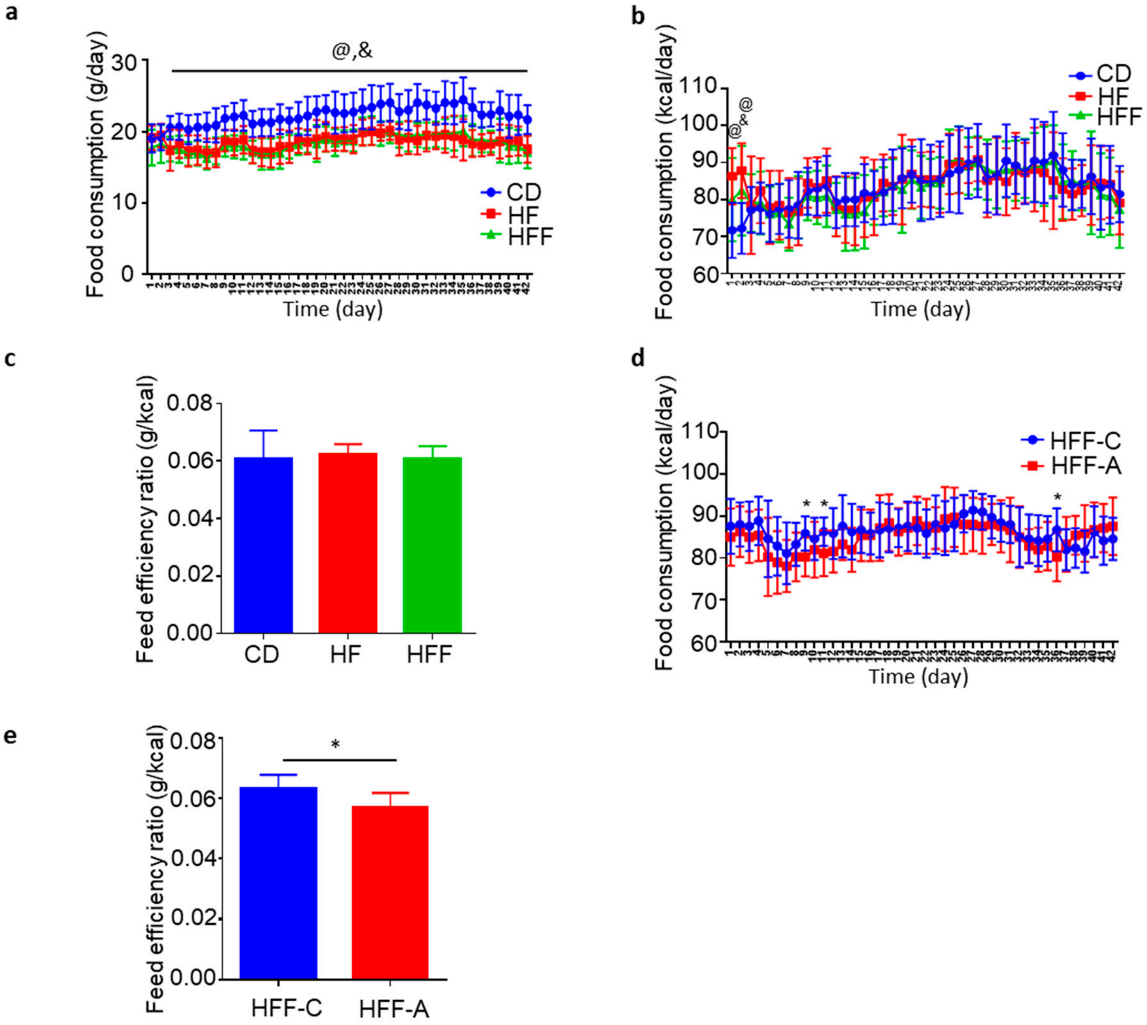

Figure S1: Food consumption (**a, b, d**) and feed efficiency ratios (**c, e**). Results are expressed as mean ± SD; @: CD vs. HF; &: CD vs. HFF. $^{@,\&}p<0.05$, $^{*}p<0.05$, **a–e**; n=10 per group. Statistical differences were determined using one-way analysis of variance (ANOVA) at each time point (**a, b**), one-way ANOVA (**c**), unpaired t-test at each time point (**d**), and unpaired t-test (**e**). CD: Control diet, HF: High-fat diet, HFF: High-fat/fructose diet, HFF-C: High-fat/fructose-cellulose, HFF-A: High-fat/fructose-D-allulose.

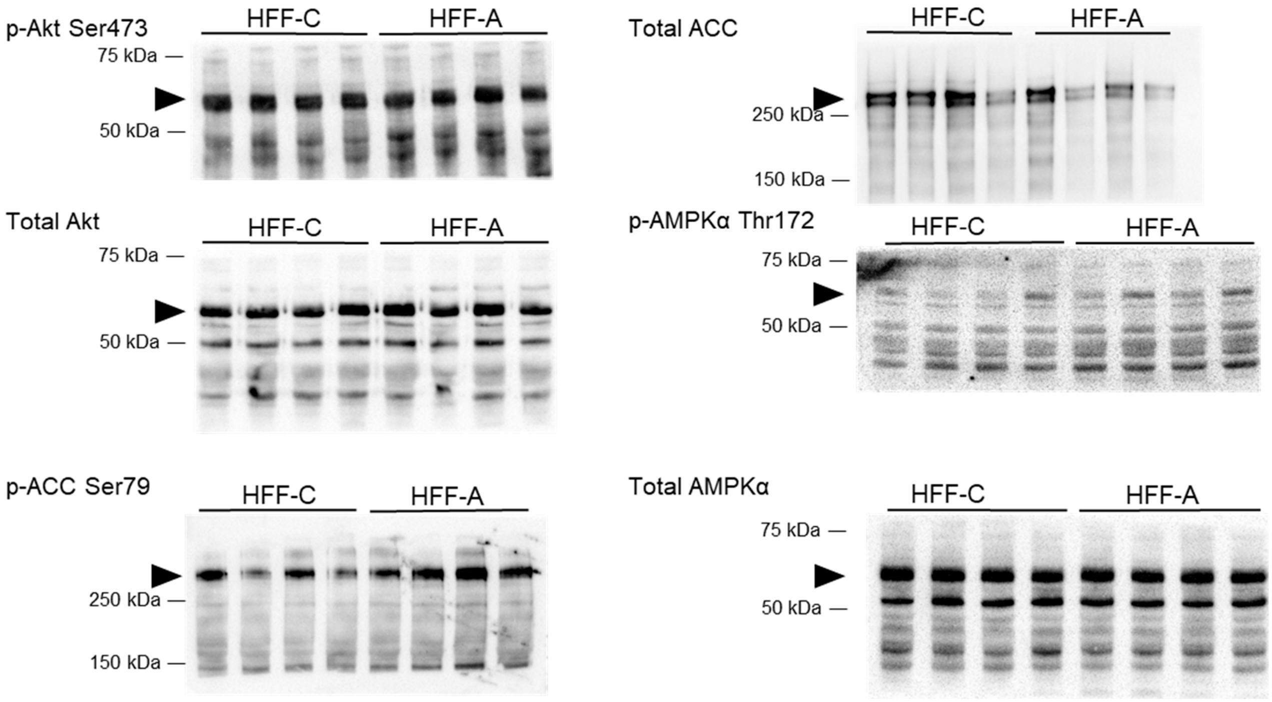

Figure S2: Uncropped images of western blots.

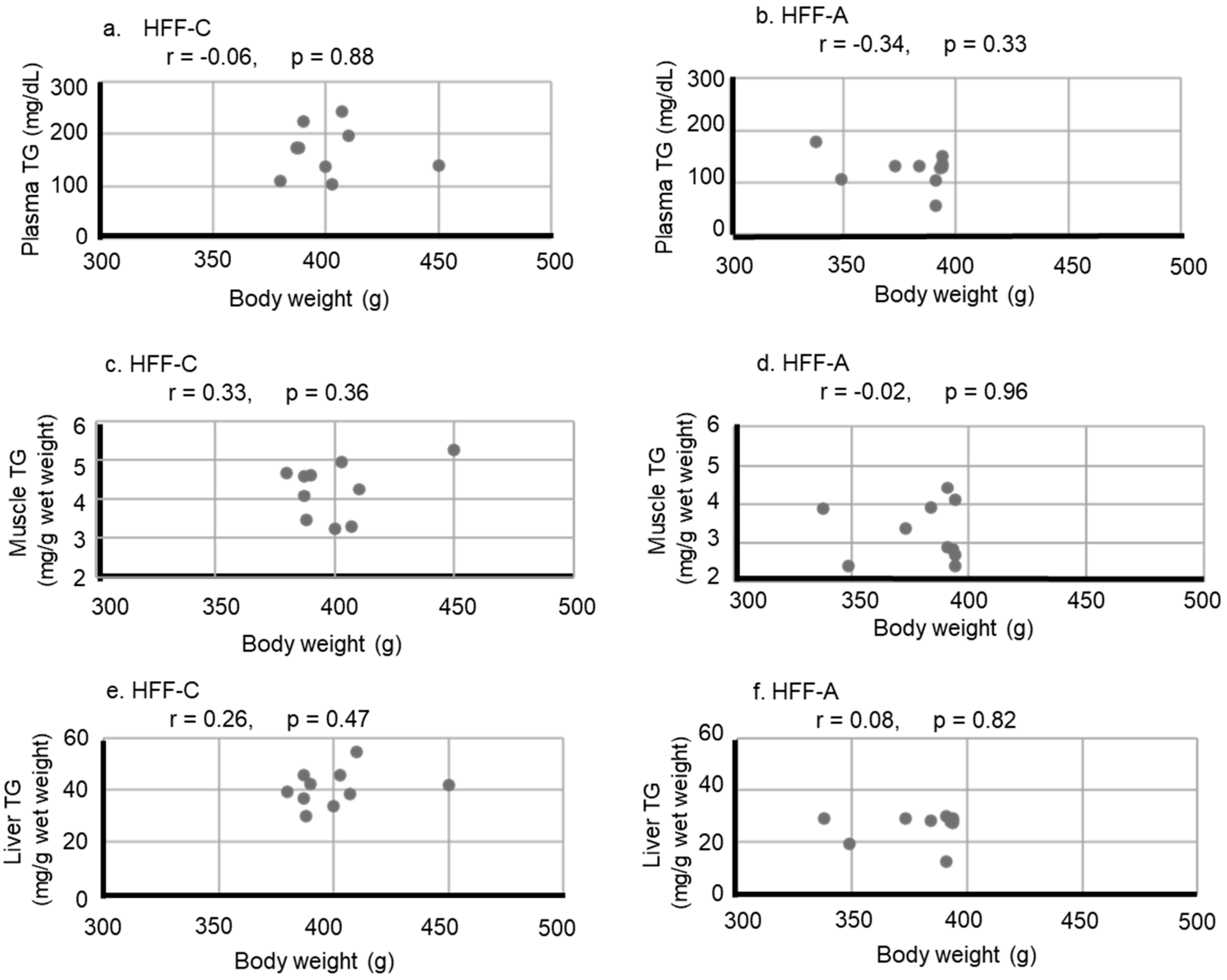

Figure S3: Relationship between body weight and TG levels in plasma, muscle, or liver for the HFF-C (**a, c, e**) and HFF-A **(b, d, f**) groups. n=10 per group. Statistical differences were determined using Pearson's correlation analysis. TG: Triglyceride, HFF-C: High-fat/fructose-cellulose, HFF-A: High-fat/fructose-D-allulose.
